# The Temporal Relationships Between Non-Suicidal Self-Injury and Social Anxiety: A Cross-Lagged Panel Study

**DOI:** 10.3390/bs16101797

**Published:** 2026-10-01

**Authors:** Yonggang Feng, Lanxiang Peng

**Affiliations:** Faculty of Education, Shandong Normal University, Jinan 250014, China; 105125@sdnu.edu.cn

**Keywords:** non-suicidal self-injury, social anxiety, rejection sensitivity, cross-lagged model

## Abstract

Empirical evidence increasingly supports an association between non-suicidal self-injury (NSSI) and social anxiety symptoms among Chinese adolescents, although their temporal relationship remains inadequately established. To address this gap, the present study employed a cross-lagged panel design to examine the longitudinal relationships between NSSI and social anxiety symptoms. The final analytic sample consisted of 614 Chinese adolescents aged 13–18 years (53.09% girls), who completed three waves of assessments at six-month intervals using standardized self-report measures of NSSI, social anxiety, and rejection sensitivity. Longitudinal analyses controlling for prior symptom levels and gender showed that social anxiety at Time 1 significantly predicted subsequent NSSI at Time 2. A reciprocal relationship between social anxiety and NSSI was also observed across subsequent measurement waves. Furthermore, rejection sensitivity showed a significant indirect effect in the longitudinal association between social anxiety and subsequent NSSI. These findings highlight the longitudinal and reciprocal associations between social anxiety and NSSI among Chinese adolescents and suggest that rejection sensitivity may represent an important psychological pathway linking social anxiety to subsequent NSSI. Intervention strategies targeting NSSI may therefore benefit from considering co-occurring social anxiety symptoms and rejection sensitivity.

## 1. Introduction

NSSI is defined as the direct and deliberate destruction of one’s own body tissue without suicidal intent (Nock, 2010). Importantly, the absence of suicidal intent during NSSI does not imply an absence of future suicide risk. Previous evidence indicates that a history of NSSI is associated with an elevated risk of subsequent suicidal thoughts and behaviors (Kiekens et al., 2018). Thus, NSSI and suicidal behavior are conceptually distinct but clinically related phenomena. It is therefore important to identify the mechanisms by which NSSI persists during adolescence, as this will help identify adolescents at risk and inform early intervention strategies.

A body of evidence supports the notion of the co-occurrence between NSSI and social problems (Peel-Wainwright et al., 2021; Neve-Enthoven et al., 2024). Within the context of negative social experience, adolescents with NSSI are likely to be highly motivated by impulsivity to obtain immediate short-term relief (Matera et al., 2021), which could in turn reinforce their social interaction problems and negative feelings. Recent evidence among college students also suggests that impulsivity may play a mediating role in the association between social anxiety and NSSI (Zou et al., 2024). For instance, the persisting anxiety symptoms related to social experiences after the single treatment for NSSI symptoms would increase the risk of relapse, as individuals with high social-emotional distress may still revert to former self-injury behaviors (Y. Wang et al., 2023). Thus, the bidirectional relationship between social anxiety and NSSI warrants our attention, particularly the potential role of impulsivity in this relationship; however, no study has explicitly investigated it yet.

Research has consistently documented the complex interconnections between non-suicidal self-injury (NSSI) and symptoms of social anxiety. From one perspective, interpersonal difficulties, particularly social anxiety, have been identified as a potential pathway through which self-injury behaviors are sustained over time. Theoretical models focusing on interpersonal aspects of NSSI propose that affected individuals might engage in self-injurious behaviors as particularly dysfunctional coping mechanisms to manage and escape from distressing emotional states (Guérin-Marion et al., 2021). Empirical evidence further reveals that people experiencing social anxiety frequently resort to NSSI as an ineffective emotion-regulation tactic (Haliczer & Dixon-Gordon, 2023). Additionally, studies demonstrate that adverse social emotions can intensify sensitivity to peer perceptions and constrict cognitive-behavioral flexibility, resulting in heightened reactivity to negative events and persistent rumination—factors that subsequently elevate the likelihood of engaging in maladaptive behaviors like NSSI (Xia et al., 2022). Conversely, self-injury may also serve as a precursor to subsequent socio-emotional impairment. More precisely, adolescents who engage in NSSI often encounter socially stressful situations, potentially due to being viewed as more emotionally vulnerable, while simultaneously experiencing intense guilt and shame—factors that collectively reinforce the stability and prolongation of social avoidance patterns (Haliczer & Dixon-Gordon, 2023). Consistent with this view, longitudinal research has established that self-injury pathology, encompassing NSSI, serves as a significant predictor of emergent social anxiety in adolescent populations.

A bidirectional relationship has been observed between social anxiety symptoms and non-suicidal self-injury (NSSI) during adolescence, though the underlying mechanisms remain inadequately elucidated (Perini et al., 2021). Rejection sensitivity (RS), conceptualized as a cognitive-affective predisposition to anxiously expect, readily perceive, and intensely respond to social rejection, may be exacerbated by social anxiety. Empirical evidence indicates that adolescents with social anxiety demonstrate accelerated detection of social rejection stimuli, which reflects a cognitive system operating in a state of hypervigilance that prioritizes the encoding of negative or rejection-related cues (Perini et al., 2021). This heightened alertness leads to the misinterpretation of neutral interpersonal information as indicative of rejection and impedes disengagement from adverse social interactions (Auyeung & Alden, 2020). It is therefore plausible that social anxiety amplifies rejection sensitivity in adolescents. Furthermore, elevated rejection sensitivity may contribute to the exacerbation of self-injurious behaviors in this population (Jiang et al., 2021). Emerging evidence suggests that high rejection sensitivity can perpetuate cycles of negative cognition and dysfunctional beliefs, thereby generating intense negative emotional states that establish the prerequisite conditions for NSSI engagement (Franssens et al., 2025). According to the rejection sensitivity model, social anxiety states readily activate threat expectations and concomitant physiological arousal (Ding et al., 2022). This heightened vulnerability to perceived rejection can precipitate adverse emotional responses and facilitate maladaptive behavioral outcomes, including self-injury (Ding et al., 2022). Consequently, a positive association is proposed between rejection sensitivity and NSSI among adolescents, with rejection sensitivity potentially mediating the relationship between social anxiety and non-suicidal self-injury.

## 2. The Current Study

The present study examined the longitudinal reciprocal associations between social anxiety symptoms and NSSI among Chinese adolescents using a three-wave cross-lagged panel design. We addressed two primary research questions. First, do social anxiety and NSSI prospectively predict one another across adolescence? Second, does rejection sensitivity represent a longitudinal mechanism linking earlier social anxiety to subsequent NSSI? In conclusion, we proposed the following hypotheses. First, higher social anxiety at an earlier wave would predict higher subsequent NSSI after accounting for prior NSSI, while higher NSSI would also predict subsequent social anxiety after accounting for prior social anxiety. Second, rejection sensitivity would show a significant indirect effect in the longitudinal association between earlier social anxiety and subsequent NSSI.

## 3. Methods

### 3.1. Participants

A cluster sampling procedure was used to recruit participants from three junior and senior high schools in Shandong Province, China. Several intact classes were randomly selected from the participating schools, and all eligible students within the selected classes were invited to participate. The sample size was determined according to the empirical rule that the number of participants should be approximately 5–10 times the number of questionnaire items (Zhang et al., 2025). Considering the three-wave longitudinal design and allowing for a potential attrition rate of approximately 30% during follow-up, a total of 650 adolescents aged 13–18 years were initially recruited. Eighteen questionnaires were excluded because of substantial missing data or invalid or inconsistent response patterns, resulting in a valid baseline sample of 632 participants. At Time 2 (T2), 625 participants completed the assessment, and 614 participants completed all three measurement waves at six-month intervals. Thus, the final analytic sample for this study consisted of 614 adolescents who completed all three measurement waves (mean age = 15.67 years, SD = 1.34), of whom 77.20% were non-only children, and 53.09% were female. No formal a priori power analysis was conducted to determine the sample size. The initial sample size was determined by the number of eligible students available in the randomly selected classes during the data-collection period. Students who transferred to another school or were absent on the day of assessment accounted for the majority of attrition. All procedures involving human participants in this study were in accordance with the ethical standards of the Academic Board of Shandong Normal University, and the 1964 Helsinki Declaration and its later amendments. Informed consent was obtained from all the participants.

### 3.2. Measures

All questionnaires were administered in Chinese.

*Non-suicidal self-injury.* NSSI was assessed with the Deliberate Self-Harm Inventory (Gratz, 2001) revised by Q. Wang and Liu (2019). The scale included nine items (e.g., “I pierce my skin with sharp objects”). The damage severity was rated on a 7-point Likert scale from 0 (none) to 6 (more than five times), and higher scores reflected higher levels of NSSI. The Cronbach’s α coefficients in this study were 0.98, 0.98, 0.99, respectively.

*Rejection sensitivity.* Rejection sensitivity was assessed with the Tendency to Expect Rejection Scale (Jobe, 2003) revised by Li (2007). The scale included 18 items (e.g., “I’m sensitive to rejection”). All items were rated on a 5-point Likert scale from 1 (Strongly disagree) to 6 (Strongly agree). Higher scores reflected higher levels of negative social bias. The Cronbach’s α coefficients in this study were 0.84, 0.80, 0.93, respectively.

*Social anxiety.* Social anxiety was assessed with the Social Anxiety Scale for Adolescents (SAS-A, La Greca & Lopez, 1998) revised by Zhou et al. (2008). The scale included 18 items (e.g., “I only talk to people I know really well”). All items were rated on a 5-point Likert scale according to how much the item “is true for you” (1 = not at all, 5 = all the time). Higher scores on this scale reflected higher levels of social anxiety. The Cronbach’s α coefficients in this study were 0.99, 0.98, and 0.99, respectively.

### 3.3. Data Analysis

Data from the final analytic sample of 614 participants were coded and entered into the database. Descriptive statistics and Pearson correlations were first calculated for the demographic characteristics and study variables. To examine the longitudinal reciprocal associations between social anxiety and NSSI, a cross-lagged panel model (CLPM) was estimated. Gender was included as a covariate in the longitudinal analyses. A longitudinal mediation model was subsequently estimated to examine the indirect effect of rejection sensitivity in the association between earlier social anxiety and subsequent NSSI. All main analyses were conducted using SPSS 26.0 and Mplus 8.6. The significance of the indirect effect was evaluated using 95% bootstrap confidence intervals based on 5000 bootstrap samples (Hayes, 2022). The de-identified dataset used in the main analyses is provided as Supplementary File S1.

## 4. Results

### 4.1. Demographic Information

Pearson correlation analysis was used to test the bivariate correlations of all the variables. Table 1 displays means, standard deviations, and bivariate correlations of all study variables. Results of the descriptive and correlation analyses are presented in Table 1; all variables at each time point had a positively significant correlation (*p* < 0.001). Harman’s single-factor analysis was used to test the common method variance effect. The total variance by one factor was less than or equal to the recommended threshold of 50% (Baumgartner et al., 2021), indicating that common method bias was not an issue for this study.

### 4.2. Reciprocal Associations Between Overparenting and NSSI

**Figure 1:**
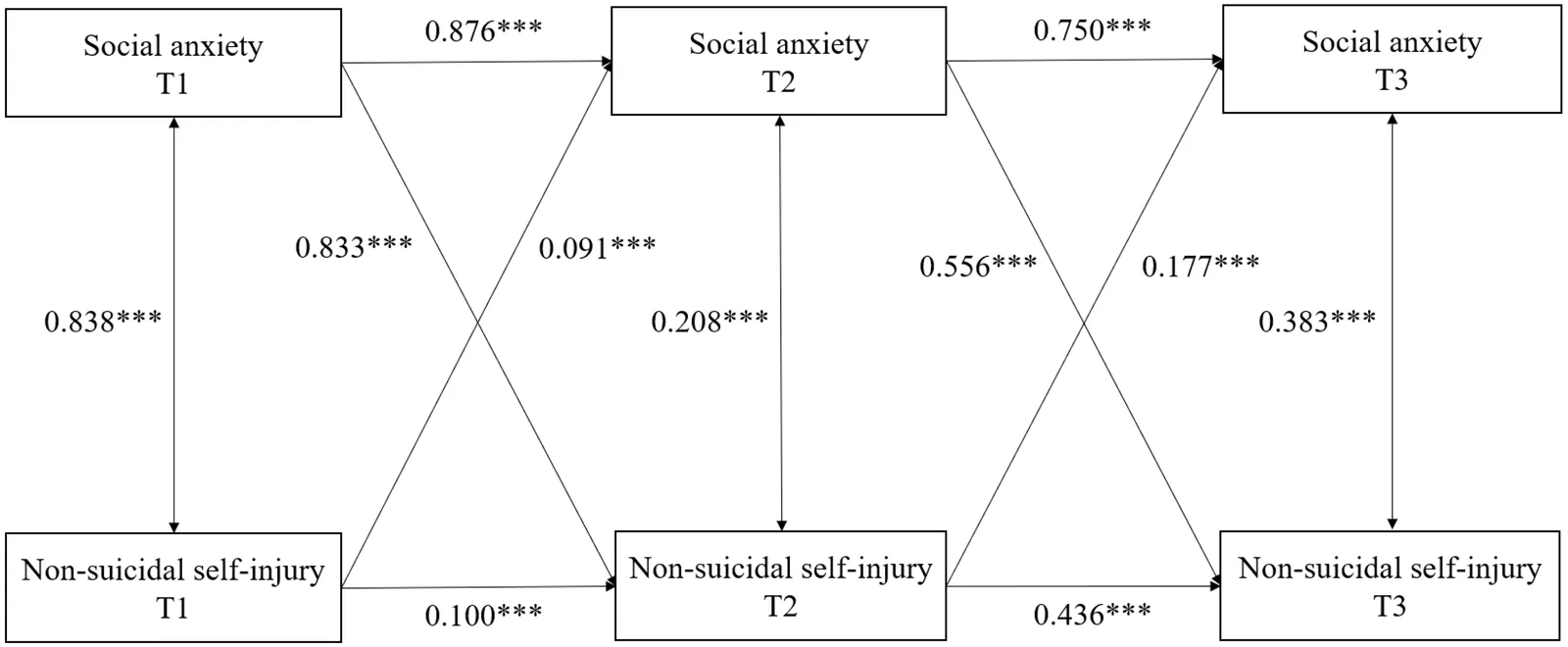
The cross-lagged panel model between social anxiety and NSSI. *** *p* < 0.001.

This study used traditional Cross-lagged Panel Models (CLPMs) to explore the interaction between social anxiety and NSSI, and the results indicate that the model fit well, with *χ*^2^ (4) = 57.84, *p* < 0.05, CFI = 0.93, and SRMR = 0.02. The cross-lagged results showed that social anxiety at Time 1 significantly predicted NSSI at Time 2, *β* = 0.833, *SE* = 0.030, *p* < 0.001; similarly, social anxiety at Time 2 significantly predicted NSSI at Time 3, *β* = 0.556, *SE* = 0.030, *p* < 0.001. NSSI at Time 1 significantly predicted social anxiety at Time 2, *β* = 0.091, SE = 0.08, *p* < 0.001; furthermore, NSSI at Time 2 significantly predicted social anxiety at Time 3, *β* = 0.177, SE = 0.045, *p* < 0.001. The results indicated that social anxiety and NSSI interacted with each other; the model is shown in Figure 1.

### 4.3. Longitudinal Mediation by Rejection Sensitivity

**Figure 2:**
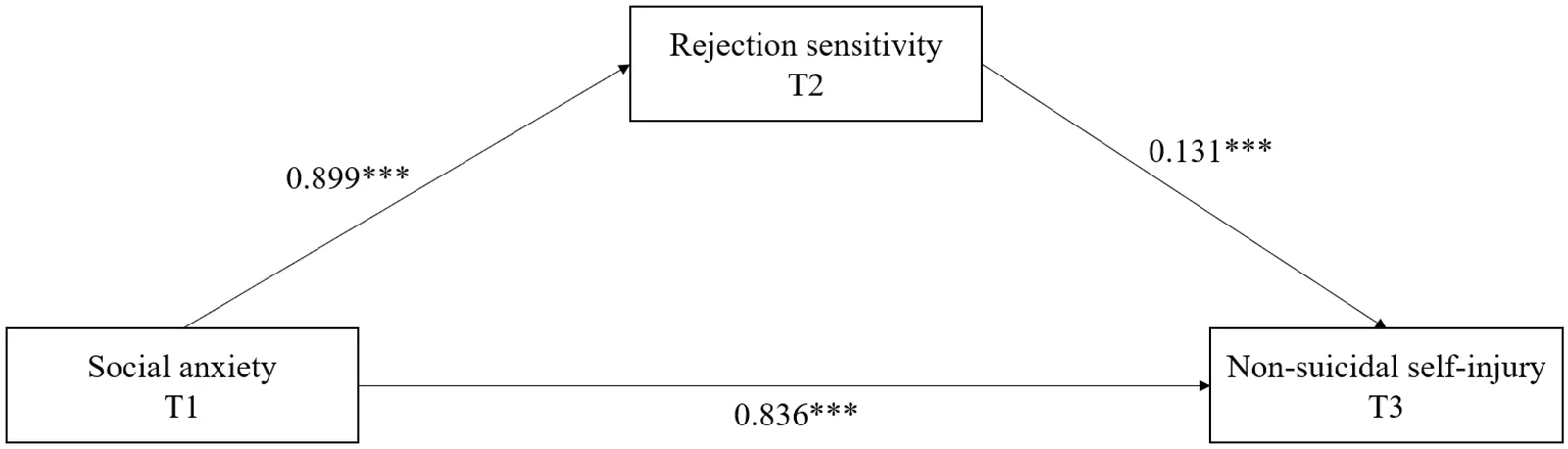
The mediating effect of rejection sensitivity. *** *p* < 0.001.

The structural equation model was used to investigate the mediating effect of rejection sensitivity, and the model was saturated. The mediation effect was estimated using the maximum likelihood estimation method. First, the mediation model with all the A paths in Figure 2 was tested. Consistent with the hypotheses, T1 social anxiety positively predicted rejection sensitivity at TTT2 (*β* = 0.899, *p* < 0.001), and positively predicted NSSI at TTTT3 (*β* = 0.836, *p* < 0.001). Rejection sensitivity at t2 positively predicted NSSI at t3 (*β* = 0.131, *p* < 0.001). The results of the bootstrap analysis revealed that social anxiety had a significant direct effect on NSSI at t3 (direct effect = 0.836, SE = 0.044, *p* < 0.001, 95% bootstrap CI [0.771, 0.917]), and a significant indirect effect on NSSI at t3 via rejection sensitivity at t2 (indirect effect = 0.118, SE = 0.042, *p* < 0.01, 95% bootstrap CI [0.039, 0.178]).

## 5. Discussion

In a longitudinal sample of Chinese adolescents, we used three waves of data to advance our understanding of the bidirectional effects between social anxiety symptoms and NSSI, as well as the mediating mechanism underlying them. Our results supported the hypothesis of a bidirectional association between social anxiety and self-injury behaviors among adolescents. The results indicated that social anxiety was a significant predictor of greater self-injury behaviors, and similar pathways existed for the longitudinal association between NSSI and social anxiety symptoms. In addition, we found that rejection sensitivity mediated the relationship between NSSI and social anxiety symptoms. Importantly, gender was included as a covariate in the longitudinal analyses, indicating that the observed longitudinal associations were estimated after accounting for gender.

Based on the cross-lagged analyses, the findings demonstrate that social anxiety symptoms and NSSI reciprocally reinforce one another across a one-year period, establishing a self-perpetuating cycle that contributes to both the initiation and persistence of self-injurious behaviors. This bidirectional pattern illuminates a key mechanism through which social stress becomes translated into repeated self-injury, progressively heightening the risk of sustained engagement in such behaviors. The observed interaction between social anxiety and NSSI provides empirical support for the defective-self model of NSSI (Hou et al., 2023), which posits that adolescent social anxiety amplifies reactivity to negative interpersonal feedback, thereby intensifying self-directed negative emotions—such as shame and guilt—and ultimately predicting the emergence of NSSI urges and actions. Consequently, it is important to recognize that within periods of heightened social anxiety, adolescents may experience significant distress from social stressors, while prior engagement in NSSI may further elevate social anxiety levels, establishing a recursive feedback loop that increases the likelihood of continued self-injury. A recent meta-analysis has corroborated this pattern, reporting strong associations between perceived rejection, interpersonal conflict, and NSSI (Cheek et al., 2020). It is also worth noting that within the Chinese cultural context, social anxiety may be particularly intensified by an emphasis on “mianzi” (face), often at the cost of genuine self-awareness, which may in turn exacerbate feelings of shame, self-criticism, and self-directed hostility.

Substantial parallels exist in the longitudinal mechanisms and theoretical models connecting NSSI and social anxiety symptoms, particularly avoidance. Empirical evidence indicates that emotional symptoms in Chinese adolescents frequently precipitate maladaptive coping responses, including self-injurious behaviors (Daukantaitė et al., 2020). These behavioral patterns subsequently establish a self-perpetuating cycle wherein negative affective states and dysfunctional coping strategies mutually reinforce one another. Consequently, this cyclical process offers a plausible explanation for the characteristically high relapse rates and treatment resistance observed in NSSI cases (Daukantaitė et al., 2020). Clinically, individuals presenting with social anxiety symptoms typically demonstrate greater psychological vulnerability, increasing their propensity to employ analogous maladaptive defense mechanisms—such as various compensatory behaviors—when confronting negative emotions, frustration, or uncertainty (Dong et al., 2022).

Also, our study shows that the effect of social anxiety on NSSI is mediated by rejection sensitivity. Simple mediation analyses indicated a significant indirect effect of social anxiety on NSSI through rejection sensitivity, which was consistent with our hypothesis. Specifically, due to being separated from peers and lacking social connection skills, adolescents might experience more rejected feelings (Schwartz-Mette et al., 2020). Studies have revealed that a lack of social connection ability is positively associated with high levels of rejection sensitivity (Jiang et al., 2021). When experiencing high levels of rejection sensitivity, adolescents try to engage in impulsive and self-injury behaviors to reduce negative emotions. These results showed that rejection sensitivity is one of the mechanisms through which social anxiety influences the frequency of self-injury behaviors. To our knowledge, this is the first study to explore the role of rejection sensitivity in the development of self-injury behaviors, indicating that interventions aimed at teaching social-emotional regulation strategies could encourage NSSI adolescents to reduce self-injury behaviors.

### 5.1. Practical Implications

Beyond enhancing theoretical comprehension of emotion-related factors in adolescent NSSI behaviors, the current results carry significant practical applications. Interventions focused on cognitive restructuring may prove particularly effective in helping adolescents reframe their interpretations of socially distressing experiences, such as perceived rejection. Educational institutions and families should implement proactive initiatives to cultivate inclusive environments that mitigate social anxiety-induced stress, thereby diminishing its influence on self-injurious behaviors. Moreover, the implementation of structured programs designed to enhance social competence could effectively redirect adolescents’ focus from emotional distress to skill acquisition. Consequently, preventative and therapeutic approaches should systematically account for the significance of interpersonal contexts in the etiology of self-injury, incorporating specific components that address adolescents’ hypersensitivity to social exclusion. Notably, considering the established function of rejection sensitivity in moderating treatment outcomes for NSSI (e.g., Selby et al., 2022), ensuring accessible psychological support and fostering socially validating environments becomes imperative for adolescents engaging in self-injurious behaviors.

### 5.2. Strengths and Limitations

This study has several limitations. Firstly, the participants were recruited from three schools in Shandong Province, China, and the sample was not designed to be nationally representative of Chinese adolescents. Therefore, the generalizability of the findings should be interpreted with caution. Future studies should include samples from other provinces in China and from different cultural contexts to further examine the generalizability of the present findings. Moreover, given that the sample in this study consists of relatively young individuals, it remains uncertain whether the findings could be generalized to broader samples. Secondly, because all focal variables were assessed using self-report measures, common method bias cannot be completely ruled out and may have inflated some of the observed statistical associations. Participants may also have overreported or underreported their responses. Thirdly, although the form of self-report in the experiments is considered reliable, investigations involving physiological indicators (e.g., heart rate and skin conductance) and neural change indicators would provide more objective and convincing evidence. Combining this with neuroimaging methods can help to better study brain state changes related to social anxiety and NSSI (e.g., Schmaal et al., 2020). Finally, only social anxiety was examined as a predictive variable in this study. Other interpersonal or environmental factors related to NSSI could be examined in future studies. Evidently, the longitudinal study design, which collected data at three time points and covered a wide range of age groups, is one of the strengths of this study. The subscale measures used to assess NSSI and social anxiety symptoms over one and a half years clearly reveal the directional and negative cycles between NSSI and avoidance symptoms. In addition, the previous literature has focused on young adults from Western countries, while the association between social anxiety and NSSI in Chinese culture has been understudied.

## 6. Conclusions

In conclusion, this investigation comprehensively delineates the dynamic longitudinal interplay between non-suicidal self-injury and symptoms of social anxiety. Furthermore, the research yields novel empirical support for theoretical frameworks of self-injurious behavior and the broaden-and-build theory by demonstrating how emotional symptomatology contributes to the maintenance of NSSI while establishing a mutually reinforcing cycle between affective disturbances and self-harm behaviors. Specifically, our analyses identified rejection sensitivity as a significant mediating pathway through which social anxiety influences the manifestation of NSSI.

## Figures and Tables

**Table 1:** Demographic and correlation description of the sample (*N* = 614).

	1	2	3	4	5	6	7	8	9	10	11	12
T1 Social anxiety	-											
T1 Rejection sensitivity	0.815 ***	-										
T1 NSSI	0.838 ***	0.549 ***	-									
T2 Social anxiety	0.953 ***	0.775 ***	0.826 ***	-								
T2 Rejection sensitivity	0.898 ***	0.646 ***	0.875 ***	0.875 ***	-							
T2 NSSI	0.917 ***	0.850 ***	0.798 ***	0.901 ***	0.775 ***	-						
T3 Social anxiety	0.941 ***	0.778 ***	0.769 ***	0.909 ***	0.862 ***	0.852 ***	-					
T3 Rejection sensitivity	0.991 ***	0.800 ***	0.888 ***	0.951 ***	0.917 ***	0.927 ***	0.928 ***	-				
T3 NSSI	0.953 ***	0.788 ***	0.856 ***	0.948 ***	0.881 ***	0.936 ***	0.917 ***	0.963 ***	-			
Gender	0.011	−0.031	0.022	0.006	0.001	−0.017	0.031	0.007	−0.013	-		
Age	0.020	0.014	0.006	0.025	0.018	0.002	0.020	0.018	0.004	0.008	-	
Only child status	0.002	0.004	0.005	−0.014	0.005	−0.001	0.010	−0.002	−0.009	0.143 **	−0.163 **	-
M	3.80	3.60	6.08	3.82	3.43	7.34	3.93	3.84	8.95	1.53	15.67	1.77
SD	1.43	0.71	6.12	1.42	0.62	6.76	1.33	0.92	0.03	0.50	1.34	0.42

Note. Gender was coded as 1 = male, and 2 = female. ** *p* < 0.01, *** *p* < 0.001.

## Data Availability

The original contributions presented in this study are included in the article. Further inquiries can be directed to the corresponding author.

## Supplementary Materials

The following supporting information can be downloaded at: https://www.mdpi.com/article/10.3390/bs16101797/s1, Supplementary File S1: De-identified dataset used for the main analyses.
